# The Murray collection of pre-antibiotic era *Enterobacteriacae*: a unique research resource

**DOI:** 10.1186/s13073-015-0222-7

**Published:** 2015-09-28

**Authors:** Kate S. Baker, Edward Burnett, Hannah McGregor, Ana Deheer-Graham, Christine Boinett, Gemma C. Langridge, Alexander M. Wailan, Amy K. Cain, Nicholas R. Thomson, Julie E. Russell, Julian Parkhill

**Affiliations:** Wellcome Trust Sanger Institute, Hinxton, CB10 1SA UK; National Collection of Type Cultures, Public Health England, Porton Down, Salisbury, SP4 0JG UK; University of Queensland, St Lucia, QLD 4072 Australia; Department of Pathogen Molecular Biology, the London School of Hygiene and Tropical Medicine, London, UK

## Abstract

Studies of historical isolates inform on the evolution and emergence of important pathogens and phenotypes, including antimicrobial resistance. Crucial to studying antimicrobial resistance are isolates that predate the widespread clinical use of antimicrobials. The Murray collection of several hundred bacterial strains of pre-antibiotic era *Enterobacteriaceae* is an invaluable resource of historical strains from important pathogen groups. Studies performed on the Collection to date merely exemplify its potential, which will only be realised through the continued effort of many scientific groups. To enable that aim, we announce the public availability of the Murray collection through the National Collection of Type Cultures, and present associated metadata with whole genome sequence data for over half of the strains. Using this information we verify the metadata for the collection with regard to subgroup designations, equivalence groupings and plasmid content. We also present genomic analyses of population structure and determinants of mobilisable antimicrobial resistance to aid strain selection in future studies. This represents an invaluable public resource for the study of these important pathogen groups and the emergence and evolution of antimicrobial resistance.

**Electronic supplementary material:**

The online version of this article (doi:10.1186/s13073-015-0222-7) contains supplementary material, which is available to authorized users.

## Background

Antimicrobial resistance (AMR) in bacteria represents a global public health crisis, and AMR in *Enterobacteriaceae* is a particularly recognised threat [1, 2]. This bacterial family includes pathogenic genera (e.g. *Salmonella*, *Escherichia/Shigella*, *Klebsiella*) that are responsible for a significant proportion of the global diarrhoeal disease burden [3] as well as systemic and nosocomial infections, often associated with heightened virulence or AMR [4, 5]. To manage these pathogens, it is critical that we understand the emergence and the evolution of clinically relevant phenotypes. Pivotal to understanding pathogen emergence and evolution is the context in which it occurred, and historical isolates have greatly informed theories regarding the emergence, disappearance and primary reservoir hosts of the pathogens that cause plague, leprosy and tuberculosis [6–8]. More recently, isolates of *Vibrio cholerae* and *Shigella flexneri* sampled from before the widespread clinical use (and consequent evolutionary pressure) of antimicrobials, i.e. the ‘pre-antibiotic’ era, were used to examine the evolution of virulence and AMR in these pathogens [9, 10]. To expand these studies in our continued efforts to understand the emergence and persistence of AMR, historical isolates must be studied alongside their contemporary counterparts.

The Murray collection (the ‘Collection’) comprises several hundred bacterial strains (mostly *Enterobacteriaceae*) collected from diverse geographic locations largely in the pre-antibiotic era (between 1917 and 1954) [11]. The Collection was amassed by the late eminent microbiologist Professor Everitt George Dunne Murray over the course of his career [12], and was stored on Douglas digest agar slopes [13]. On E.G. D. Murray’s death in 1964, the collection was passed on to his son, Robert Everitt George Murray, who was also an eminent microbiologist. In the early 1980s, R.E.G. Murray in collaboration with British microbiologists, lyophilised and transferred subcultures of the Collection from The University of Western Ontario, Canada, to the National Collection of Type Cultures (NCTC) at Public Health England, where they are held today.

Use of the Collection to provide historical context has already yielded important insights regarding the state-of-play of enteric pathogens in the first half of the 20^th^ century, and phenotypic shifts that have occurred since those times. Seminal work by scientists who coordinated the international transfer of the Collection showed that the machinery for the accumulation and plasmid-borne transfer of AMR (e.g. Incompatibility group types) [11, 14], were qualitatively similar to those of modern isolates, and this was also demonstrated for mercury resistance and *Salmonella* virulence determinants [15–17]. Other studies have demonstrated significant phenotypic shifts, including increased virulence and resistance to antimicrobials and antiseptics in *Klebsiella* sp. [18], and an increase in the magnitude and incidence of AMR in modern *Escherichia* isolates [19]. These studies however, merely exemplify the potential of the Collection. For example, its use to inform pathogen evolution through dating analyses remains entirely untapped, and enormous scope exists to further study the emergence and evolution of the pathogens, and their AMR and other traits.

In fact, the scale of the remaining work requires the coordinated expertise and effort of multiple microbiological research groups. Here, to serve that purpose, we announce the public release of the Murray collection isolates through formal accession of the 683 strains into the NCTC and provide the associated metadata. In addition to facilitating access to the physical strains, we verify the metadata by bacterial subtyping and analysis of whole genome sequencing data (also released here) generated for 370 of the strains. Finally, we present preliminary phylogenetic and gene content analyses that will aid strain selection for future scientific studies.

## Collection composition and associated metadata

The Murray collection (as held by the NCTC) comprises 683 bacterial strains belonging to 447 equivalence groups (Table 1). Equivalence groups (Additional file 1: Table S2) included strains that were related in one of the following three ways: duplicate strains in the original collection with the same name and original date; colony variants detected during subculture in Canada before transfer to the UK; or derivatives (colony variants detected during receipt of strains at NCTC). The isolates were primarily *Salmonella*, *Escherichia* and *Shigella* (which are combined here), *Klebsiella* and *Proteus* (Table 1), and fell into variably diverse subgroups e.g. subspecies, serotypes beyond those designations (see Additional file 1: Table S2; Additional file 2: Figure S1; Additional file 3: Figure S2; Additional file 4: Figure S3 and Additional file 5: Figure S4). Bacteria outside of these four main genera (see Other, Table 1) were originally poorly designated e.g. coliform, *Enterobacteriaceae*, and were subsequently determined (see ‘Confirming the collection’ below) to belong to the main genera, or the following: *Morganella*, *Rauotella*, *Aeromonas* and *Enterobacter* (Table 2, Additional file 1: Table S2).

**Table 1:** Summary of the collection contents by genus and time

	Collection			Sequenced		
	Total strains	Unique inc. equivalence groups	Years of isolation	Total strains	Unique inc. equivalence groups	Years of isolation
*Salmonella*	361	222	1917 - 1952	174	127	1917 - 1946
*E. coli/Shigella*	256	174	1917 - 1954	140	121	1917 - 1954
*Klebsiella*	42	30	1920 - 1949	35	26	1920 - 1949
*Proteus*	18	16	1919 - 1940	14	12	1919 - 1940
Other sp.	6	6	1920 - 1940	7	6	1935 - 1940
Total	683	447^a^		370	291^a^	

^a^These totals affected by an equivalence group containing both *Klebsiella* (M45) and *Escherchia/Shigella* (M162). inc. - including

**Table 2:** Assembly characteristics of the sequenced Murray collection isolates

		Assembly characteristics [mean (range)]			
Genus	No.	GC content	Total length (bp)	Contigs	N50 (bp)
*Salmonella*	174	52	4739744	44	316870
		(51–52)	(4450735–5136048)	(15–126)	(70209–992086)
*Escherichia/Shigella*	140	50	4679816	258	64933
		(50–51)	(3820214–5434207)	(63–567)	(14204–369379)
*Klebsiella*	35	56	5287110	172	117718
		(55–57)	(4980231–5582843)	(24–286)	(58784–465957)
*Proteus*	14	39	3935672	35	313856
		(38–39)	(3823752–3991064)	(18–58)	(201904–763476)
*Morganella*	3	51	3842744	23	557210
		(NA)	(3744830–3948322)	(19–29)	(403231–664661)
*Enterobacter*	2	54	5364204	58	341570
		(NA)	(5291805–5436603 )	(52–64)	(341563–341 576)
*Aeromonas*	1	59	4494408	39	166907
*Raoultella*	1	55	5488300	33	336936

**Fig. 1:**
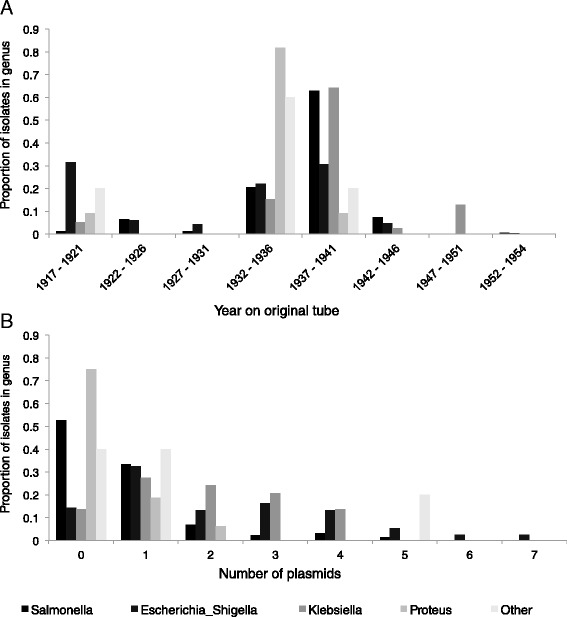
Metadata available for the Collection strains by genus, including year on original vial (**a**) and number of plasmids (**b**)

The demographic features (e.g. place, person, time) and clinical details of pathogen infection are often crucial in the interpretation of genotypic and phenotypic analyses on the isolated pathogen. Although many of these details are available for the Collection strains, this metadata is incomplete and somewhat imperfect. The diverse geographical origins of the collection “including Europe, Malta, the Middle East, northern Russia, India and North America” has been reported [11], but were not available for individual strains. Metadata held at the NCTC showed the strains originated from diverse clinical specimens, e.g. stool, urine, blood, antral washes, cerebrospinal fluid, but the clinical syndrome, e.g. meningitis, pneumonia, hepatitis and cholecystitis, or patient/supplier name were also alternatively recorded (Additional file 1: Table S2). This ‘Origin’ information was only available for approximately one quarter (n = 150) of the strains. Contrastingly however, the large majority (92 %, 628 of 683) of strains had a date or year noted on the original vial (Additional file 1: Table S2). When these dates were stratified by genus, a unique time signature emerged, perhaps reflecting E.G.D. Murray’s changing research interests over time (Fig. 1a). Notably, these dates were presumed to be the date of isolation for the strains, but could also represent date of strain receipt, or some other event. Overall however, the novel analyses presented in this study largely support the original metadata demonstrating that it is, if imperfect, robust.

In addition to the published studies on conjugative plasmids that highlighted the importance of the collection for studying mobilisable-AMR [11, 14], efforts to comprehensively determine the full plasmid content of the collection were made in the late 1980s [20]. Using traditional plasmid preparation and gel electrophoresis techniques, this work determined the number and approximate sizes of plasmids contained in each of 489 Collection strain subcultures (from [14]). The findings showed that the strains contained between zero and seven plasmids each, and that certain genera contained more plasmids than others (Fig. 1b, full results reproduced in Additional file 1: Table S2). Plasmids ranged in estimated molecular weight from 1 to 500 Md (though estimates ≥ 150Md were noted as likely to be inaccurate). Attempts to verify this plasmid content metadata among 271 strains that were also whole genome sequenced were made (see Additional file 6: Supplementary Material).

## Confirming the collection

In order to confirm the genus designations in the Collection, modern laboratory and *in silico* tools were applied to a subset of strains. The subset included all ACPD Hazard Group 2 (HG2) organisms and excluded most known HG3 organisms (23 HG3 organisms were included), thereby excluding known *Shigella dysenteriae* and *Salmonella enterica* where the serovar was unknown (see Additional file 1: Table S2). Of the total 683 isolates, 359 underwent MALDI-TOF analysis (of which 354 also underwent characterisation by 16 s rRNA sequencing). Outside of the ‘Other’ genera discussed above (and see Table 1), the MALDI-TOF results were generally concordant, with the exception of three isolates (M108, M162, M144) originally designated as *Klebsiella* that were determined to be *Escherichia/Shigella* sp., and the misidentification of a *Salmonella* isolate (M179) as an *Escherichia* by 16 s rRNA sequencing (Additional file 1: Table S2). Of the isolates that underwent MALDI-TOF analysis, 334 progressed to whole genome sequencing, alongside an additional 36 isolates not characterised by MALDI-TOF. Those revived isolates originally designated to be shigellae also underwent serotyping, and were largely confirmed (for 66 of 72 strains) to be either *S. flexneri* or *S. sonnei* as originally designated (Additional file 1: Table S2). Genus identification and *in silico* multi-locus sequence typing on whole genome sequencing data (Additional file 1: Tables S2 and Additional file 7: Table S3) confirmed the MALDI-TOF designation, or the original genus designation in all cases.

## Genomic analysis of the Murray collection

To verify the robustness of the Collection, as well as add value, provide further metadata, and facilitate the development of selection criteria for ongoing studies, 370 strains (representing 291 equivalence groups), mostly representative of the collection (Tables 1, 2, Additional file 1: Table S2 and Additional file 7: Table S3) were whole genome sequenced. Some analyses of these genomes are briefly reported here, and more detail is given in the Additional file 6: Supplementary Material.

**Fig. 2:**
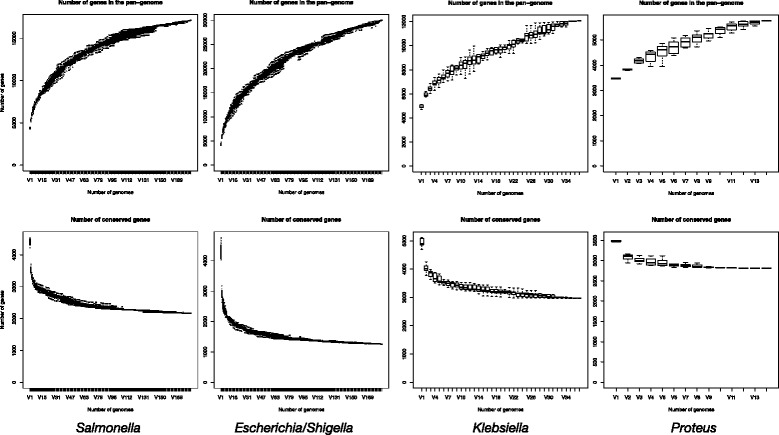
Rarefaction curves for pan- (above) and core- (below) genome sizes by genus

*De novo* assemblies created to facilitate core genome identification exemplified the unique genomic characteristics of each bacterial genus (Table 2, see Additional file 7: Table S3 for full results), which were similarly reflected in features of the core genomes including the discovery rate and final number and size of the core genome (Table 3, Fig. 2). For example, the *Proteus* had a lower GC content than the other genera (Table 2) and *Salmonella* strains had a larger core genome (Table 3) than *Escherichia/Shigella*, which had a larger accessory genome (Fig. 2).

**Table 3:** Core genome size for the main genera in the Collection

	*Salmonella*	*Escherichia/Shigella*	*Klebsiella*	*Proteus*
Total isolates (inc. refs)	185	185	37	14
Core genes (≥95 % isolates)	3002	1983	3296	2870
Core genes (100 % isolates)	2159	1255	2966	2813
Core genome (CG) length (bp)	2195115	1381269	2881098	2775840
CG variant sites (bp)	136888	114723	64138	47079

To provide enhanced subgrouping information, core genome phylogenies were constructed from the variant sites in core genes for the main genera (Additional file 2: Figure S1; Additional file 3: Figure S2; Additional file 4: Figure S3 and Additional file 5: Figure S4). In addition to providing context for future strain selection, core genome phylogenies were used to verify the designation of equivalence groups within the Collection.

## Antimicrobial resistance

**Fig. 3:**
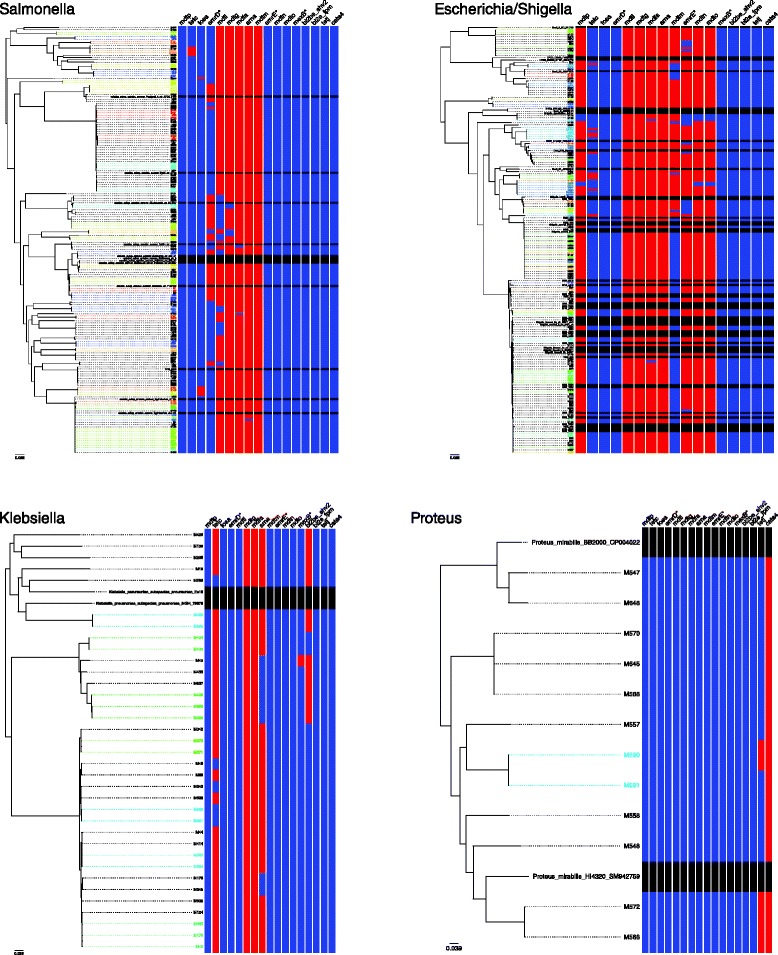
Presence (red) and absence (blue) of variably present antimicrobial resistance genes among the Collections strains overlaid adjacent to core genome phylogenies for each genus. The presence of genes in reference isolates was not determined (black)

Although no phenotypic studies of AMR were done here, AMR has been reported in the pre-antibiotic era Murray Collection strains, including tetracycline resistance in *Proteus* sp., ampicillin resistance in the *Klebsiella* and both ampicillin and kanamycin resistance in *Escherichia* sp. [11, 18, 19]. To aid the future selection of isolates based on the potential presence and absence of AMR, the presence of antimicrobial resistance genes among the strains was determined (Additional file 8: Table S1). This revealed many resistance genes (often known to be chromosomally encoded) that were present across all members of a genus, particularly across *Salmonella, Escherichia/Shigella* and *Klebsiella* whose profiles differed greatly, though unsurprisingly, from the more phylogenetically remote *Proteus.* Some genes however were differentially present among the genera with differing degrees of correlation to population structure (Additional file 8: Table S1, Fig. 3). For example, the *tetC* gene was present in nearly all *Klebsiella* isolates, but only a fraction of *Escherichia/Shigella* and *Salmonella* isolates, highlighting the potential of the Collection for studying the early horizontal transmission of AMR among *Enterobacteriaceae*.

## Summary

This study comprehensively describes a large collection of diverse bacteria (primarily *Enterobacteriaceae*) from the pre-antibiotic era, now publicly available from the NCTC, and thus represents an invaluable resource for studying the evolution and emergence of AMR and *Enterobacteriaceae*. We also created a significant genomic resource for the scientific community in the form of freely available whole genome sequencing data for over half of the strains in the Collection. Using this data, we verified much of the metadata of the Collection including species identification, plasmid content and the existence of equivalence groups among the strains. Finally, we presented additional analyses to guide future scientific studies; defining the phylogenetic subgroups and genetic determinants of mobilisable AMR present in the Collection. The availability of these live isolates, associated sequencing data and preliminary analysis to the scientific community will surely spark a spate of studies into the evolution and epidemiology of these pathogens and their antimicrobial resistances.

### Availability of supporting data

The strains in the collection are available at the NCTC under the Murray Collection Identifiers, and accession numbers shown in Additional file 8: Table S1. The whole genome sequencing data is available at the European Nucleotide Archive at (http://www.ebi.ac.uk/ena/data/view/PRJEB3255), according to the strain-specific accession numbers shown in Additional file 1: Table S2.

## Additional files

Additional file 1: Table S2. Original Collection metadata and laboratory determination of plasmid content and species. (XLSX 112 kb)

Additional file 2: Figure S1. Core genome phylogenetic tree for *Salmonella* sp. The tree is mid-point rooted. Strains noted to be in equivalence groups are similarly coloured. (PDF 39 kb)

Additional file 3: Figure S2. Core genome phylogenetic tree for *Escherichia/Shigella* sp. The tree is mid-point rooted. Reference genomes representing previously published phylogroups are indicated. Strains noted to be in equivalence groups are similarly coloured. (PDF 40 kb)

Additional file 4: Figure S3. Core genome phylogenetic tree for *Klebsiella* sp. The tree is mid-point rooted. Strains noted to be in equivalence groups are similarly coloured. (PDF 24 kb)

Additional file 5: Figure S4. Core genome phylogenetic tree for *Proteus* sp. The tree is mid-point rooted. Strains noted to be in equivalence groups are similarly coloured. (PDF 23 kb)

Additional file 6: Supplementary Material. **Table S4**. Selected references for each genus and species. **Figure S5**. Number of plasmids detected in Collection strains by laboratory and in silico approaches. Marker size is scaled by the number of strains and the trendline represents the overall correlation. (ZIP 175 kb)

Additional file 7: Table S3. Sequencing, assembly and gene content analyses for strains sequenced for this study. (XLSX 146 kb)

Additional file 8: Table S1. Antimicrobial resistance genes in sequenced strains by genus. (DOCX 66 kb)

## Abbreviations

AMR: Antimicrobial resistant/resistance
CG: Core Genome
GC: Guanine-Cytosine
MALDI-TOF: Matrix Assisted Laser Desorption Ionisation - Time of Flight
MLST: Multi-Locus Sequence Typing
NCTC: National Collection of Type Cultures
rRNA: ribosomal Ribose Nucleic Acid
UK: United Kingdom
