# Supplementary figures and images for: The Murray collection of pre-antibiotic era Enterobacteriacae: a unique research resource
Source: Genome Med. 2015 Sep 28;7:97. doi: 10.1186/s13073-015-0222-7 (PMC4584482; doi:10.1186/s13073-015-0222-7)

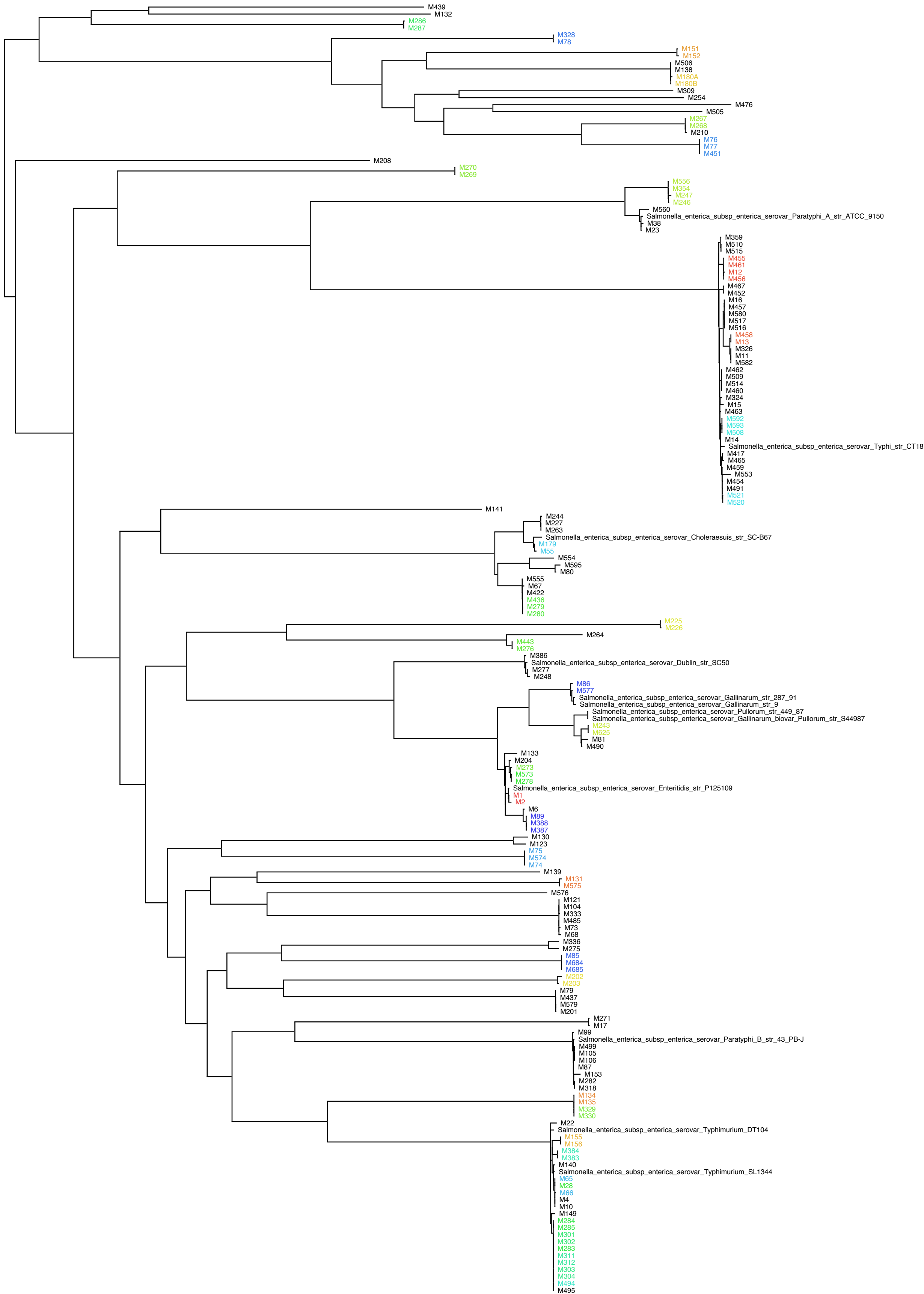

3148 SNPs

Supplement: Supplementary file 2 — Core genome phylogenetic tree for Salmonella sp. The tree is mid-point rooted. Strains noted to be in equivalence groups are similarly coloured. (PDF 39 kb) [file 13073_2015_222_MOESM2_ESM.pdf]

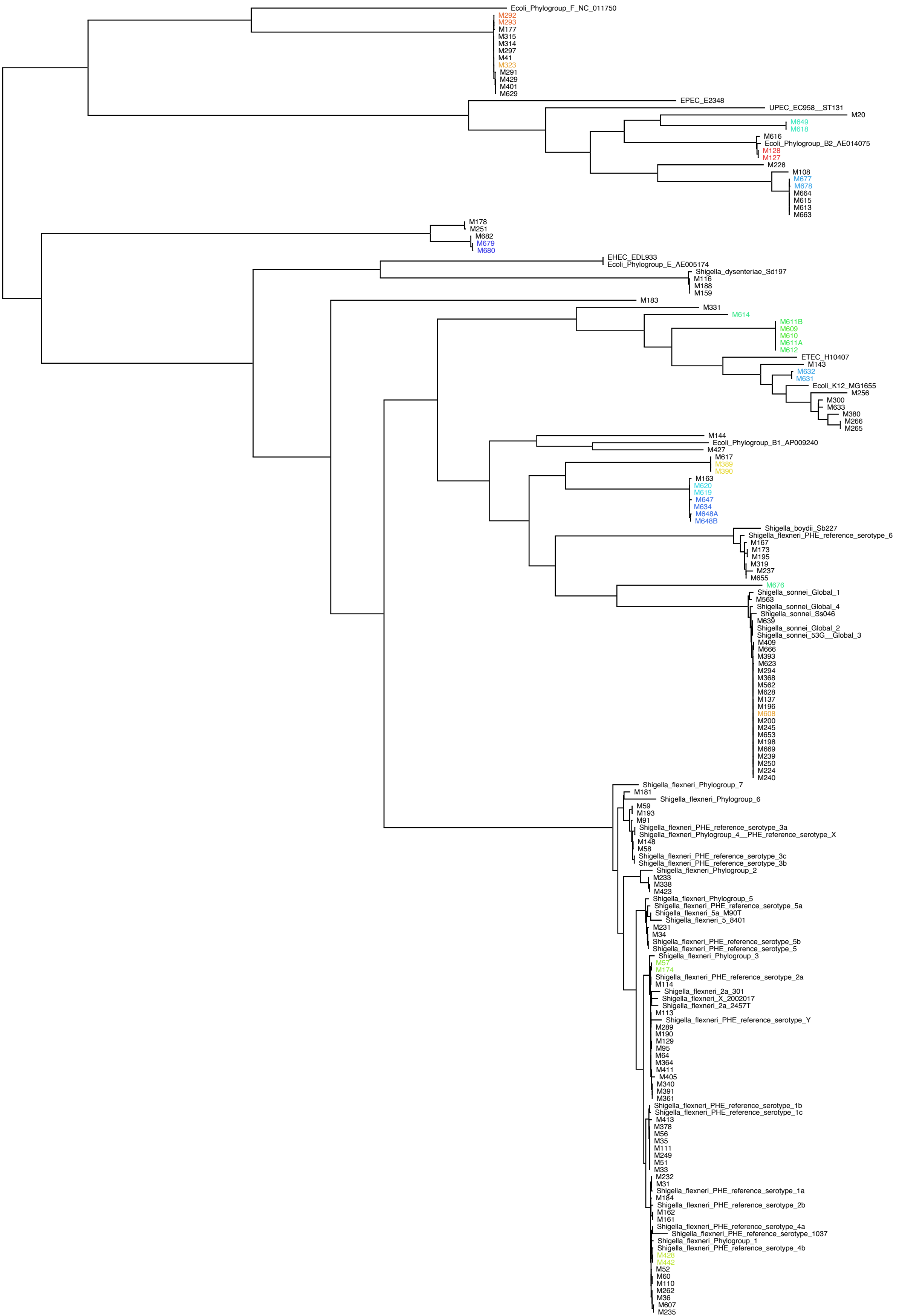

3212 SNPs

Supplement: Supplementary file 3 — Core genome phylogenetic tree for Escherichia/Shigella sp. The tree is mid-point rooted. Reference genomes representing previously published phylogroups are indicated. Strains noted to be in equivalence groups are similarly coloured. (PDF 40 kb) [file 13073_2015_222_MOESM3_ESM.pdf]

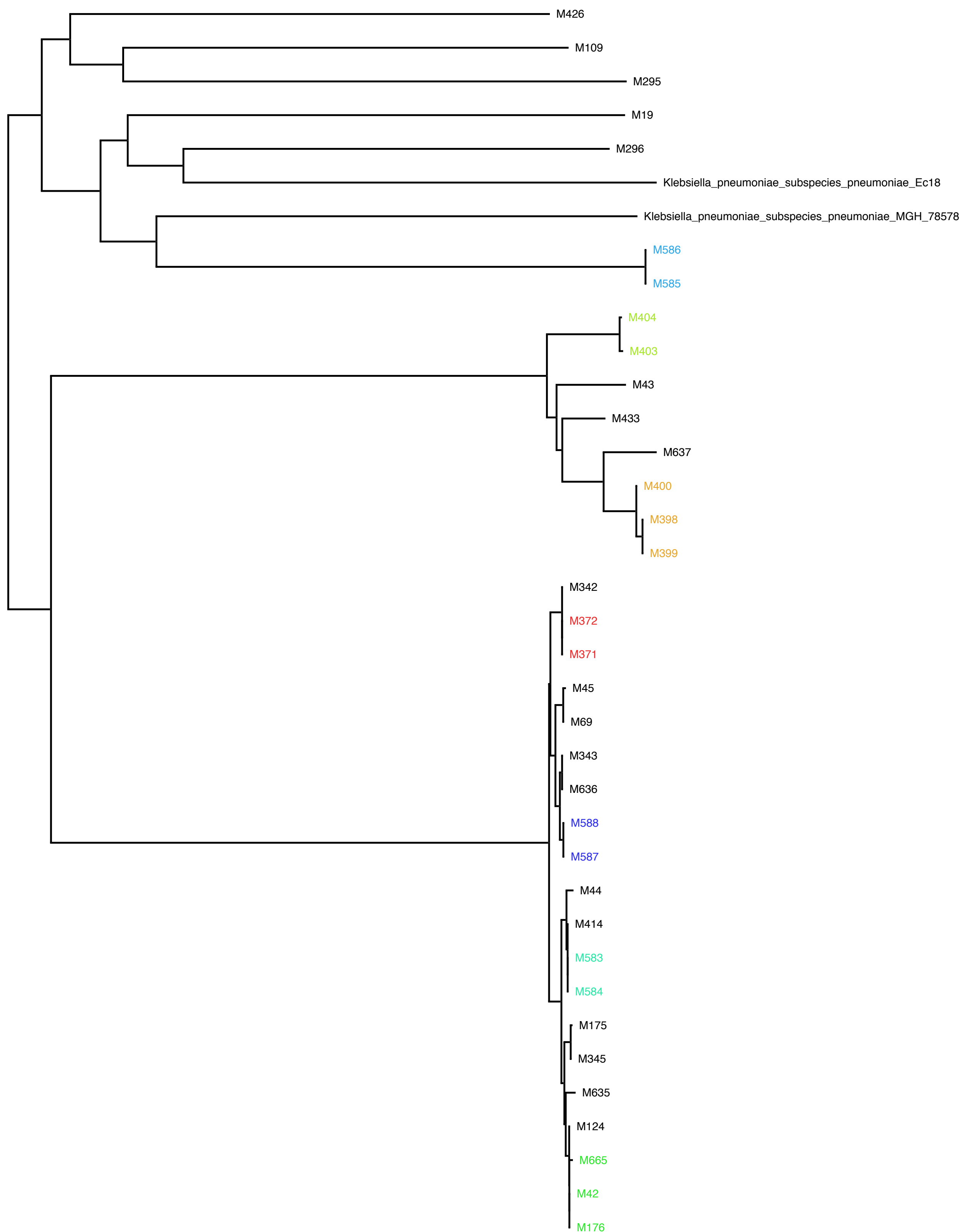

Supplement: Supplementary file 4 — Core genome phylogenetic tree for Klebsiella sp. The tree is mid-point rooted. Strains noted to be in equivalence groups are similarly coloured. (PDF 24 kb) [file 13073_2015_222_MOESM4_ESM.pdf]

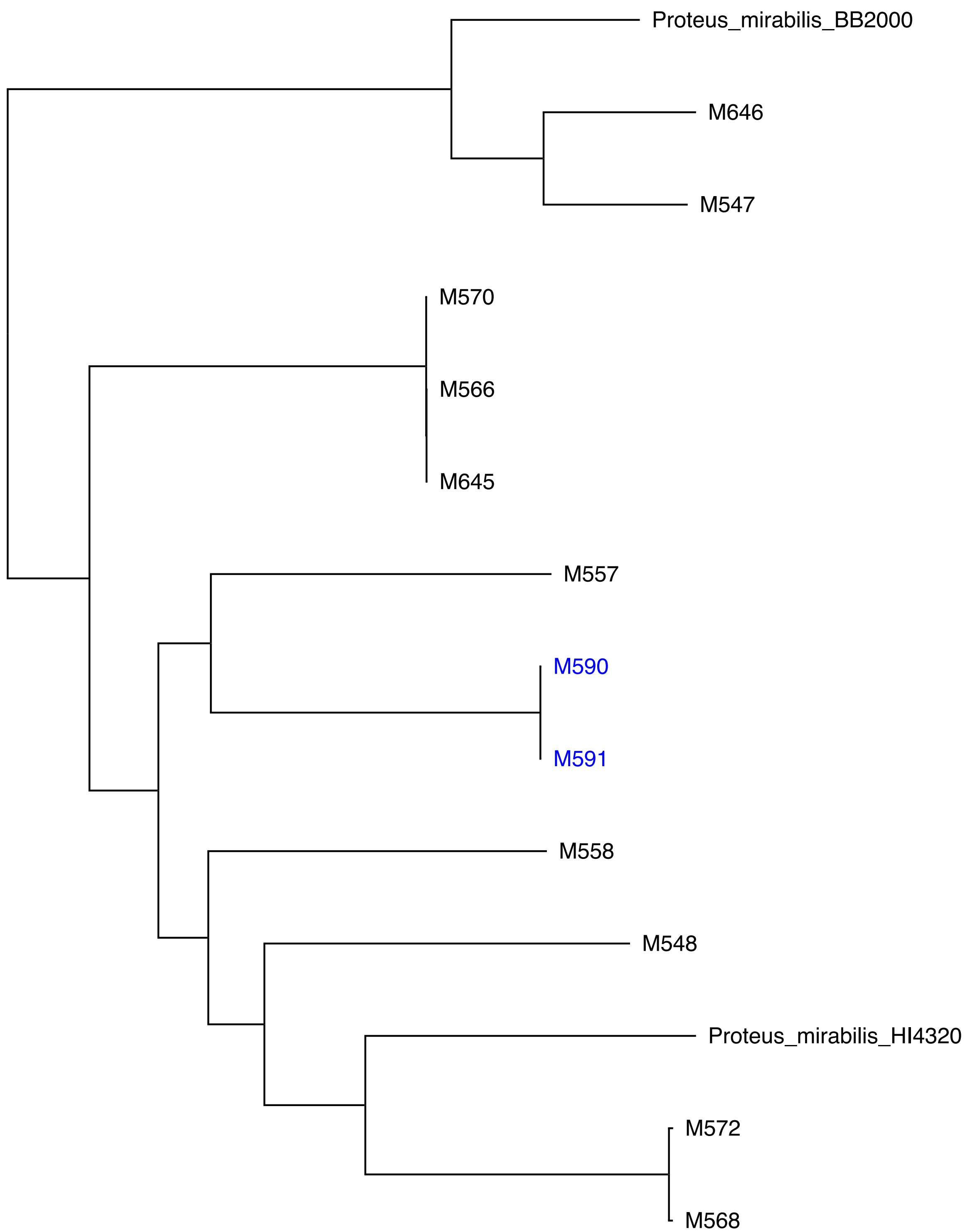

1836 SNPs

Supplement: Supplementary file 5 — Core genome phylogenetic tree for Proteus sp. The tree is mid-point rooted. Strains noted to be in equivalence groups are similarly coloured. (PDF 23 kb) [file 13073_2015_222_MOESM5_ESM.pdf]

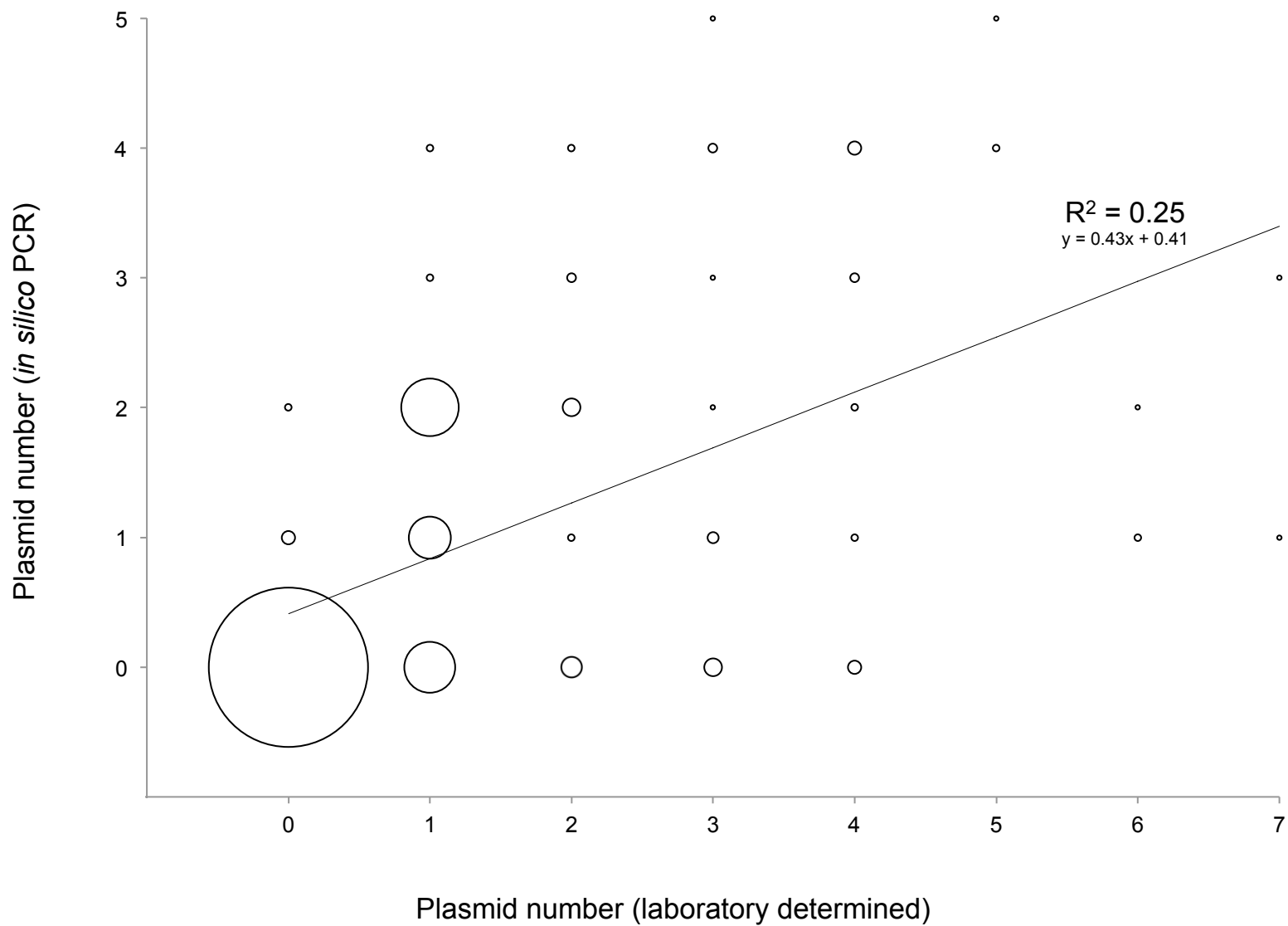

Supplement: Supplementary file 6 — Table S4. Selected references for each genus and species. Figure S5. Number of plasmids detected in Collection strains by laboratory and in silico approaches. Marker size is scaled by the number of strains and the trendline represents the overall correlation. (ZIP 175 kb) [file 13073_2015_222_MOESM6_ESM.zip › Figure S5.pdf]
