# Supplementary material for: The Murray collection of pre-antibiotic era Enterobacteriacae: a unique research resource
Source: Genome Med. 2015 Sep 28;7:97. doi: 10.1186/s13073-015-0222-7 (PMC4584482; doi:10.1186/s13073-015-0222-7)
Supplement: Supplementary file 6 — Table S4. Selected references for each genus and species. Figure S5. Number of plasmids detected in Collection strains by laboratory and in silico approaches. Marker size is scaled by the number of strains and the trendline represents the overall correlation. (ZIP 175 kb) [file 13073_2015_222_MOESM6_ESM.zip › Supplementary material.docx]

**Supplementary material for:**

**The Murray collection of pre-antibiotic era *Enterobacteriacae*: A unique research resource**

Kate S Baker^1^, Edward Burnett^2^, Hannah McGregor^2^, Ana Deheer-Graham^2^, Christine Boinett^1^, Gemma C Langridge^1^, Alexander M Wailan^3^, Amy K Cain^1^, Nicholas R Thomson^1 4^, Julie E Russell^2^, Julian Parkhill*^1^

**Address:** ^1^ Wellcome Trust Sanger Institute, Hinxton, UK, CB10 1SA; ^2^ National Collection of Type Cultures, Public Health England, Porton Down, Salisbury, UK, SP4 0JG ^3^ University of Queensland, St Lucia, Queensland, Australia, 4072; ^4^ Department of Pathogen Molecular Biology, the London School of Hygiene and Tropical Medicine, London, UK

*Corresponding author: parkhill@sanger.ac.uk

**Core genome phylogenies**

To provide further information on strain subgroups and confirm the existence of strain equivalence groups, core genome phylogenies (in the context of relevant reference genomes) were defined for the four main genera (Figures S1 – S4). With the following exceptions, results reported in this section represent the outcome for all sequenced strains (Table 1, Table S3) and selected references (detailed in Figures, Table S4) for a given genus. For *Salmonella,* M88 was excluded as preliminary analysis showed it was more closely related with *S. enterica* subsp. *arizonae* than with subsp*. enterica* (data not shown). For *Proteus,* two *P. vulgaris* strains (M549, M569) were excluded to facilitate resolution of the remaining *P.* *mirabilis* strains.

*De novo* assemblies created to facilitate core genome identification exemplified the unique genomic characteristics of each bacterial genus (Table 2, see Table S3 for full results), which were similarly reflected in features of the core genomes including the discovery rate and final number and size of the core genome (Table 3, Figure 2). For example, the *Proteus* had a lower GC content than the other genera (Table 2) and *Salmonella* strains had a larger core genome (Table 3) than *Escherichia/Shigella*, which had a larger accessory genome (Figure 2).

To provide enhanced subgrouping information, core genome phylogenies were constructed from the variant sites in core genes for the main genera (Figures S1 – S4). Notably, pre-antibiotic era representatives of many clinically important pathogen groups, including *Salmonella enterica* subsp. *enterica* serovars Typhi*,* Typhimurium and Paratyphi B (Figure S1) as well as *Shigella sonnei* and *Shigella flexneri* serotypes 1 – 5 (Figure S2) were common within the Collection. Contrastingly, representatives of more recently emerged MLST types of both *E. coli* and *K. pneumoniae* (i.e. ST131/73/95 and ST23/ST258 respectively [1] [2]) were comparatively rare (Figures S2 S3, Table S3)*.*

In addition to providing context for future strain selection, core genome phylogenies were used to verify the designation of equivalence groups within the Collection. In the case of *Salmonella* (Figure S1), 34 equivalence groups were supported by the occurrence of between 0 and 310 SNPs among members of the group, but two equivalence groups (M28/M573/M278 and M273/M330/M329) had aberrant inclusions, shown by the existence of ≥ 33,173 SNPs between M28 and M573/M278 (which differed by one SNP) and M273 and M330/M329 (which differed by 10 SNPs). Similarly, in the case of the *Escherichia/Shigella* phylogeny (Figure S2), 19 equivalence groups were supported by the presence of ≤ 218 SNPs and three groups (M614/M676, M323/M608 and M631/M632/M677/M678) were refuted by the presence of ≥ 23,529 SNPs. The latter was actually two equivalence groups M677/M678 (which differed by 63 SNPs) and M631/M632 (84 SNPs). In the case of the *Klebsiella* all 11 equivalence groups represented in the phylogeny were supported by the presence of between 2 and 167 SNPs (Figure S3) and for *P. mirabilis,* the single equivalence group was supported by no SNPs existing between the strains (Figure S4). The ‘support’ of these equivalence groups despite the occurrence of several hundred SNPs is relative considering that, in some cases, they were thought to be passages of the same strain. Further investigation is required to determine the mechanisms for these polymorphisms (e.g. long-term storage, mutation, mislabeling) and whether they genuinely underpin variation in colony morphology. Notably, some isolates that were not noted as equivalence groups in the original designations shared similar phylogenetic distances to those within an equivalence group (see Figures). The support of the vast majority of equivalence group designations however, is further support that the metadata of the Collection is robust.

**Plasmid gene content among the collection**

To verify the earlier plasmid studies and facilitate further study of mobilisable AMR in the Collection, plasmids were identified *in silico* among the sequenced strains. The diversity of incompatibility groups detected among the strains was similar to the original laboratory reports ([3], Table S3), but the number of plasmids in each strain tended to be underestimated by the *in silico* approach (though the overall correlation was poor, Figure S5, Table S3). Among the 271 isolates for which both laboratory and *in silico* determination of plasmid content were available, 342 plasmids were anticipated to be present based on laboratory findings, and only 257 (75%) were detected using the *in silico* approach (Table S2). This may have resulted from the loss of the plasmids through long-term storage, or be a consequence of database limitation, with plasmids of uncharacterised sequence being present in the Murray strains. The latter possibility is supported by the presence of a similar proportion (40 of 53, 75%) of conjugative plasmids belonging to known incompatibility groups during the studies of the Collection in the 1980s [3].

**Methods**

**Collation of Collection metadata**

All documents pertaining to the Murray collection held at the NCTC were examined and collated as part of this study.

**Laboratory methods**

For the novel analyses presented here, stocks of the Collection strains held at NCTC, lyophilized in fused glass ampoules from the 1980s were opened and reconstituted using 0.5mL nutrient broth (Public Health England, London). The reconstituted material was used to inoculate plates of Blood agar and MacConkey agar (Public Health England, London) and incubated for 18 – 24 hours at 37°C under aerobic conditions. The identity of resuscitated strains was confirmed by MALDI-TOF using the MALDI-Biotyper version 3.1 and real-time classification software version 3.4 (both Bruker DALTONIK GmbH, Bremen, Germany), using the direct-spotting method in manufacturer’s instructions. Further confirmation by 16s rRNA profiling was performed as follows. Bacterial cultures were lysed in Prepman (Applied Biosystems, Paisley, UK) and underwent PCR (Promega Mastermis, Promega, Madison, WI, USA) with ANT1F and 1392R primers (Eurofins, MWG Ebersberg, Germany). The product was purified using AMPure XP ® PCR Purification Magnetic Bead Kit (Beckman Coulter, UK) and sequenced by the Genomic Service Unit, PHE Colidale using 357F and 3R primers (Eurofins, MWG Ebersberg, Germany). The resulting sequence was compared with Genbank databases (by BLAST [4]) to obtain species identification. Isolates originally designated as shigellae also underwent rapid slide agglutination reactions performed using anti-sera for *s. sonnei* phase I and II and *S. flexneri* polyvalent 1 -6 x + y sera, according to manufacturer’s instructions (Remel, Kansas, USA).

**Bioinformatic processing**

DNA from each strain was extracted using the Wizard genomic DNA kit (Promega, Madison, WI, USA) and sequenced on an Illumina HiSeq (San Diego, CA, USA) with 150 bp paired-end reads according to in house protocols [5, 6]. Following adapter removal and quality checking, genus designations were made using Kraken [7] and high quality draft genomes were generated by *de novo* assembly (Velvet Optimiser [8]), followed by automated annotation using Prokka [9]. *In silico* MLST for *Salmonella, Escherichia/Shigella* and *Klebsiella* was performed using established schemes [10-12]. Core genomes were defined using Roary [13], which identified genes common among the Collection strains and reference strains (Table S4) that had been re-annotated as above. For each genus, individual core gene alignments were concatenated to form a core genome multiple sequence alignment and variable sites extracted to construct a maximum likelihood phylogenetic tree using RAxML [14]. Predicted protein sequences were extracted for each strain and ardbAnno [15] used to identify antimicrobial resistance genes. Plasmid sequences were identified according to covering over 98% of gene length with over 98% identity to sequences in the Plasmid Finder database [16].

**References**

1. Brisse S, Fevre C, Passet V, Issenhuth-Jeanjean S, Tournebize R, Diancourt L, Grimont P: **Virulent clones of Klebsiella pneumoniae: identification and evolutionary scenario based on genomic and phenotypic characterization.** *PLoS One* 2009, **4:**e4982.

2. Petty NK, Ben Zakour NL, Stanton-Cook M, Skippington E, Totsika M, Forde BM, Phan MD, Gomes Moriel D, Peters KM, Davies M, et al: **Global dissemination of a multidrug resistant Escherichia coli clone.** *Proc Natl Acad Sci U S A* 2014, **111:**5694-5699.

3. Datta N, Hughes VM: **Plasmids of the same Inc groups in Enterobacteria before and after the medical use of antibiotics.** *Nature* 1983, **306:**616-617.

4. Altschul SF, Gish W, Miller W, Myers EW, Lipman DJ: **Basic local alignment search tool.** *J Mol Biol* 1990, **215:**403-410.

5. Quail MA, Kozarewa I, Smith F, Scally A, Stephens PJ, Durbin R, Swerdlow H, Turner DJ: **A large genome center's improvements to the Illumina sequencing system.** *Nat Methods* 2008, **5:**1005-1010.

6. Quail MA, Otto TD, Gu Y, Harris SR, Skelly TF, McQuillan JA, Swerdlow HP, Oyola SO: **Optimal enzymes for amplifying sequencing libraries.** *Nat Methods* 2012, **9:**10-11.

7. Wood DE, Salzberg SL: **Kraken: ultrafast metagenomic sequence classification using exact alignments.** *Genome Biol* 2014, **15:**R46.

8. Zerbino DR, Birney E: **Velvet: algorithms for de novo short read assembly using de Bruijn graphs.** *Genome Res* 2008, **18:**821-829.

9. Seemann T: **Prokka: rapid prokaryotic genome annotation.** *Bioinformatics* 2014.

10. Achtman M, Wain J, Weill FX, Nair S, Zhou Z, Sangal V, Krauland MG, Hale JL, Harbottle H, Uesbeck A, et al: **Multilocus sequence typing as a replacement for serotyping in Salmonella enterica.** *PLoS Pathog* 2012, **8:**e1002776.

11. Wirth T, Falush D, Lan R, Colles F, Mensa P, Wieler LH, Karch H, Reeves PR, Maiden MC, Ochman H, Achtman M: **Sex and virulence in Escherichia coli: an evolutionary perspective.** *Mol Microbiol* 2006, **60:**1136-1151.

12. Diancourt L, Passet V, Verhoef J, Grimont PA, Brisse S: **Multilocus sequence typing of Klebsiella pneumoniae nosocomial isolates.** *J Clin Microbiol* 2005, **43:**4178-4182.

13. Page AJ, Cummins CA, Hunt M, Wong VK, Reuter S, Holden MTG, Fookes M, Keane JA, Parkhill J: **Roary: Rapid large-scale prokaryote pan genome analysis.** *bioRxiv* 2015.

14. Stamatakis A: **RAxML-VI-HPC: maximum likelihood-based phylogenetic analyses with thousands of taxa and mixed models.** *Bioinformatics* 2006, **22:**2688-2690.

15. Liu B, Pop M: **ARDB--Antibiotic Resistance Genes Database.** *Nucleic Acids Res* 2009, **37:**D443-447.

16. Carattoli A, Zankari E, Garcia-Fernandez A, Voldby Larsen M, Lund O, Villa L, Moller Aarestrup F, Hasman H: **In silico detection and typing of plasmids using PlasmidFinder and plasmid multilocus sequence typing.** *Antimicrob Agents Chemother* 2014, **58:**3895-3903.
