# Supplementary material for: The Murray collection of pre-antibiotic era Enterobacteriacae: a unique research resource
Source: Genome Med. 2015 Sep 28;7:97. doi: 10.1186/s13073-015-0222-7 (PMC4584482; doi:10.1186/s13073-015-0222-7)
Supplement: Supplementary file 8 — Antimicrobial resistance genes in sequenced strains by genus. (DOCX 66 kb) [file 13073_2015_222_MOESM8_ESM.docx]

**Table S1. Antimicrobial resistance genes in sequenced strains by genus**

|  | Proportion of isolates containing gene | | | |
| --- | --- | --- | --- | --- |
| Antibiotic Resistance Gene | *Salmonella* | *Escherichia/Shigella* | *Klebsiella* | *Proteus* (inc. *P. vulgaris*) |
| *bl3_cpha* | 0.00 | 0.00 | 0.00 | 0.00 |
| *catA2* | 0.00 | 0.00 | 0.00 | 0.00 |
| *bl2e_fpm* | 0.00 | 0.00 | 0.00 | 0.14 |
| *tetJ* | 0.00 | 0.00 | 0.00 | 0.21 |
| *catA4* | 0.00 | 0.00 | 0.00 | 0.86 |
| *fosA* | 0.03 | 0.00 | 0.00 | 0.00 |
| *ermD** | 0.25 | 0.00 | 0.00 | 0.00 |
| *pbp2* | 1.00 | 0.00 | 0.00 | 0.00 |
| *mexB** | 0.00 | 0.00 | 0.03 | 0.00 |
| *bl2be_shv2* | 0.00 | 0.00 | 0.37 | 0.00 |
| *tetC* | 0.02 | 0.07 | 0.86 | 0.00 |
| *mdtM* | 1.00 | 0.44 | 0.00 | 0.00 |
| *emrE** | 0.00 | 0.87 | 0.00 | 0.00 |
| *mdtN* | 0.00 | 0.96 | 0.00 | 0.00 |
| *mdtO* | 0.00 | 0.96 | 0.00 | 0.00 |
| *mdtP* | 0.01 | 0.96 | 0.00 | 0.00 |
| *mdfA* | 0.99 | 0.99 | 1.00 | 0.00 |
| *bl1_ec* | 0.00 | 1.00 | 0.00 | 0.00 |
| *mdtE* | 0.00 | 1.00 | 0.00 | 0.00 |
| *mdtF* | 0.00 | 1.00 | 0.00 | 0.00 |
| *mdtL* | 0.83 | 1.00 | 0.00 | 0.00 |
| *arnA* | 0.99 | 1.00 | 0.77 | 0.00 |
| *mdtG* | 0.97 | 1.00 | 1.00 | 0.00 |
| *acrA* | 1.00 | 1.00 | 1.00 | 0.00 |
| *acrB* | 1.00 | 1.00 | 1.00 | 0.00 |
| *bacA* | 1.00 | 1.00 | 1.00 | 0.00 |
| *bcr* | 1.00 | 1.00 | 1.00 | 0.00 |
| *ksgA* | 1.00 | 1.00 | 1.00 | 0.00 |
| *macB* | 1.00 | 1.00 | 1.00 | 0.00 |
| *mdtH* | 1.00 | 1.00 | 1.00 | 0.00 |
| *mdtK* | 1.00 | 1.00 | 1.00 | 0.00 |
| *tolC* | 1.00 | 1.00 | 1.00 | 0.00 |
| Total isolates (number) | 174 | 140 | 35 | 14 |
| * dependencies not met; these genes act with others, not all of which are present. | | | | |
